# Citrus tristeza virus: A large RNA virus with complex biology turned into a valuable tool for crop protection

**DOI:** 10.1371/journal.ppat.1008416

**Published:** 2020-04-30

**Authors:** Svetlana Y. Folimonova

**Affiliations:** University of Florida, Plant Pathology Department, Gainesville, Florida, United States of America; University of Michigan Medical School, UNITED STATES

## Introduction

**Fig 1:**
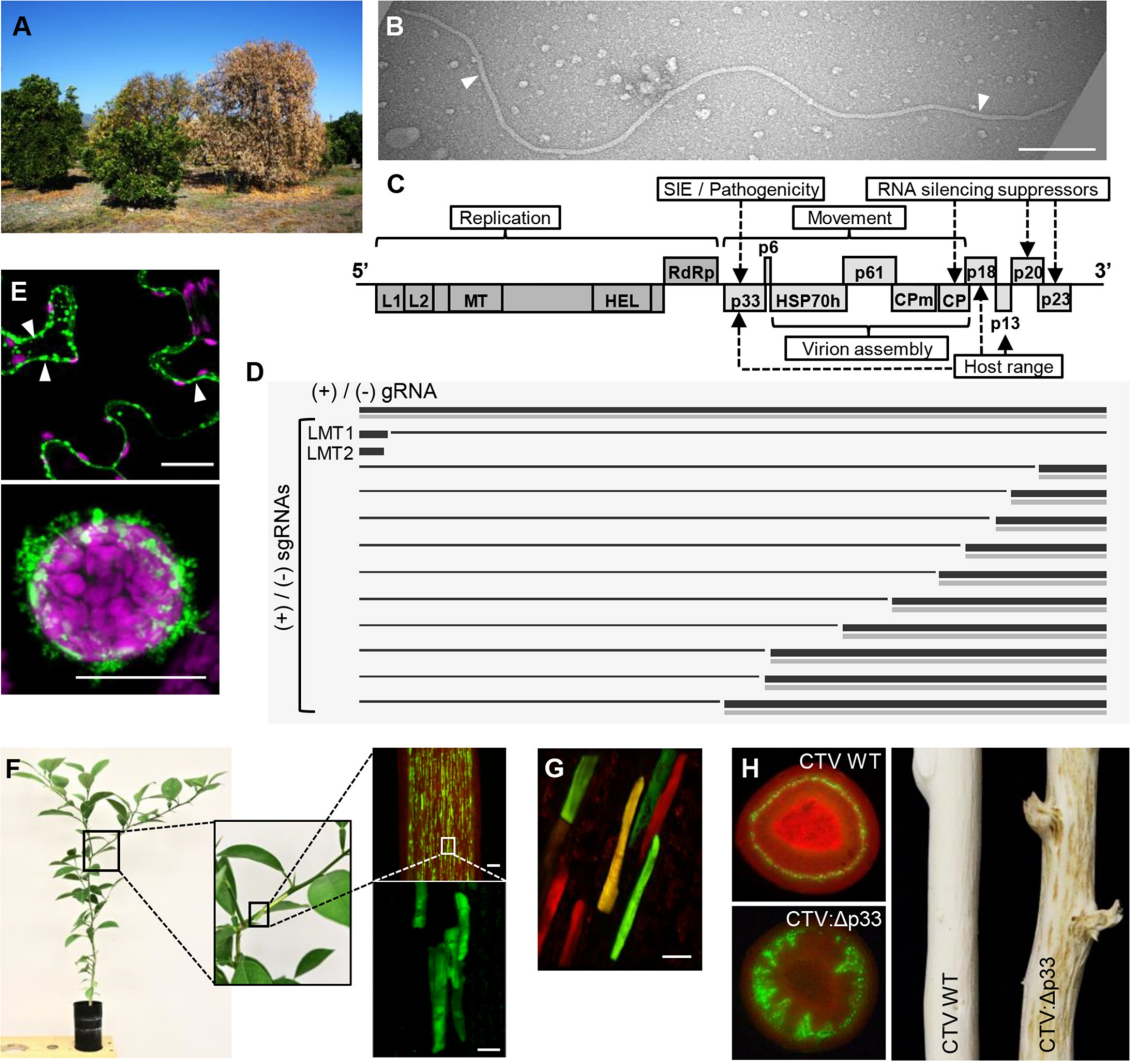
CTV traits. (A) A field citrus tree showing decline. (B) CTV virion. Image was captured by transmission electron microscopy. White arrowheads point to the virion. Bar, 200 nm. (C) Schematic diagram of the CTV genome organization. The open boxes represent ORFs and their translation products. (D) RNA species produced upon replication and expression of the CTV genome. Black and grey lines indicate positive-stranded and negative-stranded genomic and sg RNAs, respectively. (E) GFP-tagged p33 expressed in *Nicotiana benthamiana* cells forms punctate spots at plasmodesmata (top image, white arrowheads) and exhibits the ability to form extracellular tubules (bottom image). Images show confocal projection (top image) and a confocal single section (bottom image) of a *N*. *benthamiana* cell (top image) or protoplast (bottom image), which were visualized along with the autofluorescence of the chloroplasts (purple color). Images were taken at 4 days after the introduction of the corresponding expression cassettes. Scale bars, 20 μm. (F) Observation of fluorescence in phloem-associated cells on the internal surface of bark from a citrus seedling infected with the GFP-tagged CTV at 3 months after inoculation. Images were taken using a dissecting fluorescence microscope (top image on the right) and a confocal laser scanning fluorescence microscope (bottom image). Bars, 0.5 mm and 20 μm, respectively. (G) Observation of fluorescence in phloem-associated cells of a citrus tree superinfected with two nonexcluding CTV variants, each expressing an extra gene of GFP or of a red fluorescent protein. Yellow cell is coinfected with both variants. Image was taken at 12 weeks after challenge inoculation using a confocal fluorescence microscope. Bar, 20 μm. (H) Tissue distribution and symptoms produced by the GFP-tagged CTV WT or a p33 deletion mutant (CTV:Δp33) in a citrus tree at 12 months after inoculation. Images on the left were taken using stem cross-sections and a dissecting fluorescence microscope. Red color in the left images results from chlorophyll autofluorescence. CP, major coat protein; CPm, minor coat protein; CTV, citrus tristeza virus; GFP, green fluorescent protein; HEL, helicase-like domain; HSP70h, HSP70 homolog; L1, L2, papain-like leader protease domains; LMT1, low-molecular-weight tristeza RNA 1; LMT2, low-molecular-weight tristeza RNA 2; MT, methyltransferase-like domain; ORF, open reading frame; RdRp, an RNA-dependent RNA polymerase; sg, subgenomic; SIE, superinfection exclusion; WT, wild type.

Citrus tristeza virus (CTV), a member of the family Closteroviridae, represents one of the most intricate viruses with an overwhelmingly complex biology. From a standpoint of economic importance, CTV is regarded as the most destructive viral pathogen of citrus. During the past century, virus-induced epidemics of quick decline (Fig 1A), a disease, which was named “tristeza” after the Portuguese word for “sadness,” killed millions of trees in the citrus-growing areas in North and South America and the Mediterranean [1, 2]. These days, the virus still continues to threaten the citrus industries in many different regions around the world. From a research standpoint, CTV is one of the most challenging viruses to handle, which is due to the large size of its RNA genome, the fragile nature of the virions that have a shape of long flexuous thread-like filaments (Fig 1B and Fig 1C), and a narrow host range limited to slow-growing *Citrus* species in which the virus primarily infects phloem-associated cells. Despite all these difficulties, there have been many instances when research on CTV has resulted in the discovery of novel aspects and phenomena in virus–virus and virus–host interactions and has allowed to generate reagents, which may not be amenable with small, less complex viruses. This Review discusses five exciting and important facts about this virus.

## CTV is among viruses with largest RNA genomes

CTV possesses a 19.3 kilobase (kb) positive-stranded RNA genome, which is nearly twice as large as the average sized RNA virus genome of 10 kb [3]. In the world of plant viruses, CTV stands out as the virus with the largest nonsegmented RNA genome. In a broader perspective, the size of the CTV genome could be compared with that of animal nidoviruses whose genomes are represented by positive-sense RNA molecules ranging between 20 and 41.1 kb in size [4]. Besides the size, the genome of CTV that encodes 12 open reading frames (ORFs) has other peculiar features, among which are the presence of two sets of duplicated and diverged genes, e.g., the genes for the papain-like leader proteases and the genes for the major and minor coat proteins as well as the presence of a gene closely related to the genes coding for the cellular heat shock proteins in the HSP70 family and of three genes coding for suppressors of host RNA silencing [3, 5] (Fig 1C). The most 5’-proximal ORF, ORF1a, is expressed from the genomic RNA resulting in production of a 349-kDa protein that contains a tandem of two leader protease domains plus methyltransferase-like and helicase-like domains. Translation of ORF1a occasionally continues into ORF1b encoding the polymerase-like domain by a frameshift in an unusual +1 configuration. It was suggested that the large polyproteins undergo further proteolytic processing mediated by the viral proteases, which releases protein subunits participating in virus replication in a manner similar to other closteroviruses [6, 7]. The other 10 ORFs are expressed via 3’-coterminal subgenomic RNAs (sgRNAs) and encode proteins involved in assembly of CTV virions, virus movement, suppression of the host antiviral response, and expansion of the viral host range [5] (Fig 1C and Fig 1D).

Most of the CTV proteins play multiple roles in the virus infection cycle, which is in agreement with often found multifunctional nature of viral proteins. Yet, one of them, a unique nonconserved p33, appears to be the champion when it comes to the functions it performs. The research demonstrated that p33 is a membrane-associated protein, and its membrane association confers virus ability to extend the host range [5, 8]. The p33 protein possesses certain characteristics found in a number of movement proteins of other plant viruses: It localizes at plasmodesmata (the channels within the cell walls of plant cells connecting the cytoplasm of adjacent cells and enabling transport of materials) and has the ability to polymerize forming extracellular tubules [9] (Fig 1E). Furthermore, p33 is a key factor in CTV superinfection exclusion (SIE; this phenomenon is discussed below in more detail) and also is a viral effector that influences virus pathogenicity by modulating a host immune response [10–12].

## CTV produces a myriad of virus-specific RNA species in the infected cells

During infection, CTV produces a plethora of different RNA species. Those include the genomic RNA and its complementary negative-stranded copy, a nested set of 3’-coterminal sgRNAs that serve as mRNAs for translation of the internal and 3’-end genes and their corresponding negative-stranded complements along with the 5’-terminal positive-stranded sgRNAs, which are generated as a result of promoting or terminating activity of the same sgRNA controller elements (CEs; [1]; Fig 1D). With CTV, the mechanism of sgRNA production is not fully understood, and, thus, the RNA signatures that drive their synthesis are referred to as CEs, rather than promoters or terminators. There are also two abundant 5’-terminal positive-stranded noncoding sgRNAs of about 750 and 650 nucleotides in size [1]. Those were named as low-molecular-weight tristeza RNAs 1 and 2 (LMT1 and LMT2, respectively). Not much is known about the shorter RNA, besides an observation that the emergence of LMT2 correlates with virus assembly [13]. On the other hand, LMT1 whose production is driven by a specific 5’ CE is crucial for CTV infection. LMT1 counteracts the host response against the invading virus by subverting the phytohormone-mediated (e.g., salicylic acid-mediated) plant defense [14]. In addition to these sgRNAs, various defective RNAs composed of variable-in-size 5’- and 3’-sequences with or without sequences derived from the central genomic region and large amounts of small RNAs generated in the process of viral RNAs being targeted by the host RNA silencing machinery add to the complexity of the RNA species produced upon CTV infection [2, 5, 15].

## Peculiarity of the interactions between CTV variants

CTV has numerous variants, which could be grouped in at least seven major strains. Strains of CTV are defined as phylogenetically distinct lineages, which are classified based upon analysis of the viral genomic sequences [2]. Individual viral samples, on the other hand, are designated as isolates. Remarkably, it is not possible to link a CTV strain to a specific phenotype: Virus variants within the same strain could induce very different phenotypes, with some causing severe disease and others being mild or symptomless. The development of CTV-related disease syndromes such as quick decline, stem pitting, or seedling yellows depends on the infecting virus variant as well as on the citrus rootstock and scion combination. The degree of this complexity is further elevated by the fact that, in the field, long-living citrus trees are often infected by mixtures of variants of several different strains, which have been brought up by vectoring insects (e.g., aphids). Thus, a virus isolate obtained from an infected tree could be a population of multiple strains [2]. The overall phenotype or a degree of its manifestation would depend on the composition and structure of a particular virus population, which results in a wide range of phenotypes that can be found.

The explanation for coexistence of multiple strains of CTV in a host came from research on CTV SIE. SIE, a phenomenon in which a primary virus infection prevents a secondary infection with the same or closely related virus, has been observed with viruses in various systems, including important pathogens of humans, animals, and plants. With CTV, SIE only occurs between virus variants of the same strain, while variants belonging to different strains do not exclude each other, permitting formation of multistrain populations in the same host [16] (Fig 1F and Fig 1G). With variants of the same strain, SIE functions as a powerful mechanism that completely prevents superinfection of the host plant by another variant. Furthermore, the phenomenon could not be explained by the mechanisms described previously for other viruses. It is operated at two different levels—the whole organism and the cellular level—and is mediated by multiple viral factors: the viral p33 protein and a factor(s) encoded in the 5’-terminal part of the CTV genome, which is still under investigation [10, 11, 17].

Knowledge obtained from the research on CTV SIE can be translated into practical applications that would allow protection of citrus trees in the field from aggressive CTV variants. One such application is crossprotection or preimmunization, a purposeful inoculation of trees with mild virus variants. This strategy has been widely practiced in many citrus-growing regions, yet with low success due to the lack of understanding of the mechanisms behind a mild variant-mediated protection [18]. Knowing that the protecting inoculum has to carry a CTV variant of the same strain as the disease-causing component in the severe isolate could make selection of protecting isolates more efficient [19].

## Virus tissue tropism revisited

Traditionally, CTV has been considered as a phloem-limited virus [2, 5]. On the other hand, a recent study examining a virus mutant lacking the p33 protein demonstrated that the mutant can be often found replicating in the immature xylem tracheid cells. Spread of the p33 deletion variant beyond phloem and its invasion into the xylem correlated, on one hand, with a weaker host response compared with that to the full-length wild-type virus and, on the other hand, with abnormal vascular tissue differentiation and the development of stem pits [12] (Fig 1H). The findings of this study suggested that phloem restriction of CTV results from the outcome of the CTV-plant host arms race and could be influenced by factors modulating the host immune response. It appears, though, that keeping the virus within the phloem is beneficial for both sides as it allows accumulation and long-lasting persistence of the virus without the detrimental damage to the host.

## CTV as a tool for crop protection

A few years ago, CTV was developed into a vector for transient expression of sequences of interest (e.g., genes of polyproteins or RNA fragments) in citrus trees, which opened new avenues for managing citrus and, possibly, other perennial crops [20]. Upon infection of citrus trees, this vector was capable of expressing the foreign marker gene continuously for many years [20]. Fortunately, there are many variants of CTV that do not cause disease in most citrus cultivars. Those would be good candidates for the development of expression vectors.

Several important traits make the CTV-based vector an attractive tool for crop management. One of them is its unprecedented stability: the modified virus has retained and expressed a foreign insert in planta for years, which compares to days or weeks for most other vectors engineered based on smaller plant viruses. Such a feature of the CTV vector correlates with an unusual intrinsic stability of the CTV consensus genome and slow evolutionary rate shown for this virus [20]. The CTV vector could be used to screen candidate genes for potential beneficial functions in citrus or as vehicles to express known sequences or silence host genes to protect or modify existing trees. Currently, the CTV vector is being tested as a tool to manage citrus greening (known as Huanglongbing [HLB]), a devastating bacterial disease that has been endemic in most citrus-growing areas in Asia for a long time and more recently has spread throughout citrus regions in South America and then in North America [20]. The variants of the vector carry potentially protective sequences (e.g., genes of antimicrobial polypeptides or RNA interference sequences) that are designed to target Candidatus *Liberibacter asiaticus*, the disease causal agent, or the insects that transmit the bacterium. Importantly, although expected to work for a relatively long term, the modified viruses will eventually lose the inserted sequences such that those vectors would not add anything permanently to the environment.
